# Glucocorticoids and physical performance: A systematic review with meta-analysis of randomized controlled trials

**DOI:** 10.3389/fspor.2023.1108062

**Published:** 2023-03-23

**Authors:** Amund Riiser, Trine Stensrud, Lars Bo Andersen

**Affiliations:** ^1^ Faculty of Teacher Education, Art and Sport, Western Norway University of Applied Sciences, Sogndal, Norway; ^2^ Department of Sport Medicine, Norwegian School of Sport Sciences, Oslo, Norway

**Keywords:** doping, endurance, strength, sprint, corticosteroids

## Abstract

**Introduction:**

This systematic review with meta-analysis investigates the effect of glucocorticoids on maximal and submaximal performance in healthy subjects.

**Methods:**

We searched for randomised controlled trials investigating the effect of glucocorticoids on physical performance in Web of Science, Scopus, Medline, Embase and SportDiscus in March 2021. Risk of bias was assessed with the revised Cochrane Collaboration Risk of Bias Tool (RoB2). Data from random effect models are presented as standardized difference in mean (SDM) with 95% confidence interval. We included 15 studies comprising 175 subjects.

**Results:**

Two studies had high risk of bias. Glucocorticoids had a small positive effect on maximal physical performance compared to placebo (SDM 0.300, 95% CI 0.080 to 0.520) and the SDM for the 13 included comparisons was not heterogeneous (*I*2^ ^= 35%, *p* = 0.099). Meta regression found no difference in the effect of acute treatment vs. prolonged treatment or oral ingestion vs. inhalation (*p* > 0.124). In stratified analysis prolonged treatment (SDM 0.428, 95% CI 0.148 to 0.709) and oral ingestion (SDM 0.361, 95% CI 0.124 to 0.598) improved physical performance. Glucocorticoids improved aerobic performance (SDM 0.371, 95% CI 0.173 to 0.569) but not anaerobic performance (*p* = 0.135). Glucocorticoids did not change energy expenditure during submaximal performance (SDM 0.0.225 95% CI −0.771 to 0.112).

**Discussion:**

This study indicates that glucocorticoids improves maximal performance and aerobic performance. Glucocorticoids did not affect the energy expenditure during submaximal performance. The conclusions are based on relatively few subjects leading to limited statistical power and uncertain estimates. Still, these results are consistent and should be of interest to WADA and anyone concerned about fair play.

**Systematic Review Registration:**

Open Science Framework 2021-04-29 (https://osf.io/fc29t/).

## 1. Introduction

Glucocorticoids are anti-inflammatory drugs used to treat medical conditions such as asthma (1) and musculoskeletal inflammatory conditions (2). Asthma (3) and musculoskeletal inflammation are common conditions in athletes and sports medicine physicians regularly prescribe glucocorticoids for elite athletes (4).

However, athletes have been suspected to use glucocorticoid to improve athletic performance since the 1960s (5). Glucocorticoids may enhance physical performance through several pathways including increased availability of metabolic substrates through increased lipolysis (6), proteolysis (7), and availability of glucose (5). Glucocorticoids may also have immunosuppressive and anti-inflammatory effects that may prevent the immune system from overreacting as a result of exercise-induced muscle damage (8), and cortisol seems to prepare the organism for the next bout of exercise (9). In addition, glucocorticoids may stimulate cerebral glucocorticoid receptors leading to reduced muscle pain during exercise, raised fatigue threshold and positive hedonic responses, which may translate to increased physical performance (10). Glucocorticoids have a high potential for adverse effects including muscle waste, and these effects depends multiple factors like type of glucocorticoid, duration of treatment, dose and route of administration (11). Thus, athletes taking glucocorticoids to improve performance probably prefers shorter periods of administration.

The World Anti-Doping Association (WADA) annually update its prohibited list, a list of substances and methods prohibited in elite sports. The prohibited list, effective from January 1st, 2022, prohibits all glucocorticoids in competition when they are administered by any oral, rectal or injectable route, as these administration forms are considered to have a systemic effect. Other routes of administration like inhalation topical application and local injections are approved in competition as the are considered to have less potential for improving performance. All use of glucocorticoids out of competition is approved (12). To help physicians treat athletes and comply with the anti-doping rules WADA has from 2022 recommends specific wash out periods (time from the last dose to the day before competition) for different types glucocorticoids and administration route. From 2022 WADA also introduced specific urinary reporting levels for different types of glucocorticoids as different glucocorticoids has large variation in elimination time (13).

The first randomized controlled trials (RCTs) investigating the effect of glucocorticoids on high intensity exercise were published in the middle of the 1990s (14, 15). They investigated the effect of glucocorticoids on heart rate and oxygen consumption during high intensity running intervals. Since then several RCTs have investigated the effect of glucocorticoids on submaximal and maximal physical performance. As the modes of administration, type of glucocorticoid, duration of treatment, dose, population, and exercise protocol may vary between the trials, they reach different conclusions regarding the effect of glucocorticoids on physical performance, however it is mainly studies investigating the effect of high oral doses administered over time on endurance cycling to exhaustion that shows effect. The physical performance and metabolic effect of glucocorticoids on healthy subjects have previously been reviewed (5, 10), but without a systematic search of the literature and the results from the included studies are not combined in statistical analysis. Thus, we aim to systematically review and meta-analyse RCTs investigating the effect of glucocorticoids on maximal or submaximal performance in healthy subjects.

## 2. Methods

### 2.1. Search strategy and selection criteria

#### 2.1.1. Literature search

Two university librarians systematically searched for RCTs examining the effect of glucocorticoids on exercise performance in healthy humans on 25th of March 2021. Studies reporting submaximal or maximal exercise performance were eligible. Peer reviewed RCTs published in English were identified from five electronic databases Web of Science, Scopus, Medline, Embase and SportDiscus. The search consisted of three blocks (Healthy Volunteers OR athletes) AND (glucocorticoid OR betamethasone OR beclomethasone OR budesonide OR ciclesonide OR cortisone OR cortivazol OR deflazacort OR dexamethasone OR diflucortolone OR flunisolide OR fluticason OR hydrocortisone OR methylprednisolone OR mometason OR prednisolone OR prednisone OR triamcinolone acetonide) AND (exhaustion OR power OR endurance OR strength OR aerobe* OR anaerobe* OR exercise OR athletic performance). The search strategy was adopted to each database.

The full search strategy for Ovid MEDLINE(R) (Epub Ahead of Print, In-Process, In-Data-Review & Other Non-Indexed Citations and Daily <1946 to March 24, 2021>) was “(1) exp Healthy Volunteers/(20634) (2) health*.ti,ab. (2898020) (3) exp Athletes/(14209) (4) athlete*.ti,ab. (54558) (5) (non* adj1 asthma*).ti,ab. (2893) (6) 1 or 2 or 3 or 4 or 5 (2953011) (7) exp Glucocorticoids/(195417) (8) (glucocorticoid* or betamethasone* or beclomethasone* or budesonide* or ciclesonide* or cortisone* or cortivazol* or deflazacort* or dexamethasone* or diflucortolone* or flunisolide* or fluticason* or hydrocortisone* or methylprednisolone* or mometason* or prednisolone* or prednisone* or triamcinolone acetonide*).ti,ab. (216858) (9) 7 or 8 (307391) (10) exp Athletic Performance/(56265) (11) (exhaustion or power or endurance or strength or aerobe* or anaerobe* or exercise or athletic performance).ti,ab. (902259) (12) 10 or 11 (925075) (13) (random* and (controlled or control or placebo or versus or vs. or group or groups or comparison or compared or arm or arms or crossover or cross-over) and (trial or study)).ti,ab. (711495) (14) ((single or double or triple) and (masked or blind*)).ti,ab. (195072) (15) exp Randomized Controlled Trials as Topic/(144879) (16) exp Randomized Controlled Trial/(526321) (17) 13 or 14 or 15 or 16 (1027594) (18) 6 and 9 and 12 and 17 (173) (19) limit 18 to English (169)” For full search strategy in Web of Science, Scopus, Embase and SportDiscus see Supplementary Table S1.

**Figure 1:**
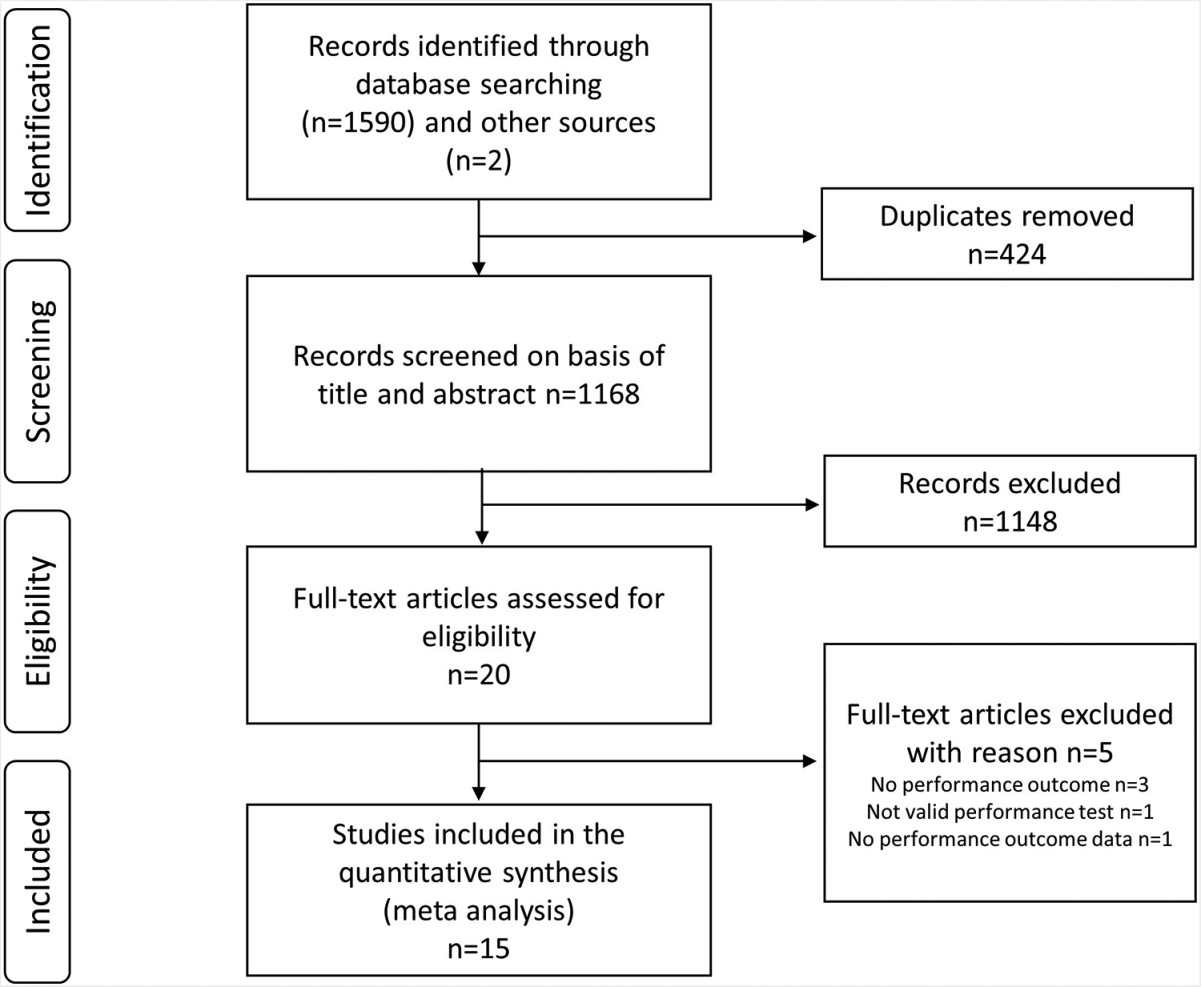
Flow chart of included studies as proposed by preferered reporting items for systematic reviews and meta-analyses statement 2020.

The search identified 1,606 records (Web of Science 305, Scopus 615, Medline 83, Embase 567, SportDiscus 20 records). After elimination of duplicates, 1,166 records remained, and these were supplemented with two additional records that the first author had previous knowledge of (Figure 1). The first author also performed backward and forward citations screening of reviews on the topic (5, 16) the 20th of Marts 2021. Twenty studies were selected for full-text eligibility assessment after screening of titles and abstracts. We excluded one study examined the effect of glucocorticoids on submaximal performance while adjusting load according to heart rate. We excluded that study as we do not consider the test to be a valid performance test (17), three other studies were excluded as they had no performance outcome and one study was excluded as it presented no data on the performance outcome (Figure 1).

#### 2.1.2. Inclusion criteria and the selection process

Two authors (AR and LBA) independently screened the studies 1,168 records on basis of title/abstract for eligibility and assessed 20 full text articles for eligibility. Discrepancies were resolved by discussion. We included RCTs with healthy humans/athletes examining the effect of glucocorticoids on physical performance. There was no restriction regarding type of glucocorticoid, administration mode, duration of treatment or dose. Both studies examining physiological parameters such as heart rate and oxygen consumption during fixed submaximal exercise or maximal endurance performance were included, in addition to studies assessing maximal endurance, strength, speed and power outcomes.

### 2.2. Analysis

Two authors (AR and TS) extracted data independently from all included studies and discrepancies in collected data were resolved by consensus. All performance test data were collected, also if one study presented data from multiple performance tests. We included only one outcome in each analysis, and we prioritized the outcomes most similar to athletic competitions and with shortest duration. The performance test involving the most muscle mass was selected (chest press was selected over biceps curl and handgrip), high intensity/short duration outcomes were selected over outcomes with lower intensities/longer duration. For example, 30 m sprint was selected over 20 m shuttle-run and one leg kicking to exhaustion, kicking to exhaustion over ∼110 s was selected over kicking to exhaustion over ∼350 s, cycling sprint was selected over cycling time trail, and maximal force was selected over time to exhaustion during hopping. Aerobic performance was defined as maximal performance tests (time trial or time to exhaustion) lasting more than 1 min and anaerobic performance was considered maximal performance tests lasting less than 1 min (18). In three-way crossover studies the effect of both active interventions (compared with placebo) were included in the meta-analysis. One study compared maximal force in four consecutive 30 s hopping intervals (19), and we meta-analysed the four trials into one result and used it in further meta-analysis. When we analysed the effect of glucocorticoids on submaximal performance, we meta-analysed the results from high and low responders to dexamethasone (measured as plasma adrenocorticotropic hormone) and defined increased kilo calorie expenditure during 1 h cycling and increased VO_2_ at iso load as increased energy expenditure. In addition to outcomes, results and interventions (acute: treatment administred at on timepoint and prolonged: treatment administred over several days), design (crossover, three-way crossover or parallel), the number of subjects included in the analysis, gender, age and any description of the subject’s fitness level were collected. Correlation between performance with glucocorticoids and with placebo was not reported in the included studies, thus a correlation coefficient of 0.5 was imputed for all comparisons. Standard deviation (SD) was computed when standard error of the mean (SEM) was reported in the included studies. Outcome data were collected as mean with SD/SEM post glucocorticoid treatment and placebo for crossover trials and as mean with SD/SEM pre and post glucocorticoid treatment and pre and post placebo for parallel trials. We sent email to the corresponding author for the study not reporting outcome data after glucocorticoids and placebo (just stating “not modified by the treatments”) and requested data for use in the meta-analysis (20) but the data were not available. Thus, we excluded the study.

### 2.3. Study quality assessment

The included studies were assessed using the revised Cochrane Collaboration Risk of Bias Tool (RoB2) (21) to evaluate five standard domains plus one extra domain for crossover trials. According to the tool criteria each domain was scored as low risk of bias, some concerns or high risk of bias. Overall risk of bias for each study was determined by the highest risk of bias across all domains. Two authors (AR and LBA) independently assessed the included studies and discrepancies in the assessment were resolved by discussion.

### 2.4. Statistics

We performed meta-analysis with random effects models as we deemed the included studies to be heterogeneous with regards to interventions, outcome measures and populations. We used Comprehensive Meta-Analysis (CMA) V3 (Biostat, Englewood, New Jersey, United States) to perform meta-analysis. In the main and subgroup analysis, only one outcome was included in each meta-analysis also if one study reported multiple outcomes. Effect estimates are presented as standardised difference in mean (SDM) with 95% CI and in forest plots. Heterogeneity is reported as *I*2 and *p*-values. Whether the effect of glucocorticoids was related to duration of treatment or routes of administration was analysed by meta regression (test of model). We also performed stratified analysis on acute and prolonged treatment, and oral ingestion and inhalation. As sensitivity analysis we also analysed the effect of glucocorticoids on maximal performance excluding the studies with high risk of bias. Potential publication bias was assessed by funnel plots, Begg and Mazumdar rank correlation test and classic fail-safe. The adequacy of sample size in each included study was assessed by calculation of the sample size required to obtain an alfa of 0.05 and a beta of 0.2 (22). Skewness in the outcomes was assessed by baseline mean/SD, and a baseline mean/SD > 2 was considered to be skewed (23). We performed sensitivity analysis excluding the studies with high risk of bias and the studies with less than 10 data pairs. Significance level was set to *p* < 0.05.

## 3. Results

### 3.1. Study characteristics

In total 15 studies were included in the systematic review with meta-analysis comprising 25 different comparisons of performance between glucocorticoids and placebo. The studies included 147 subjects in crossover trials and 28 subjects in a parallel trial. Thirteen studies assessed the effect of glucocorticoids on maximal performance (19, 24–34) and 3 studies assessed the effect of glucocorticoids on submaximal performance (14, 35, 36). For characteristics of the included studies see Table 1.

**Table 1:** Characteristics of the included studies.

Study, year	Design	Subjects: *n*, sex, age years ± SD	Fitness	Intervention	Outcome
Arlettaz et al., 2008	Crossover	14, m, 25 ± 10	Cycling and/or running 2–3 times/week for at least 3 years	Oral prednisolone 20 mg	Cycling to exhaustion at 70%–75% of VO_2max_
Arlettaz et al., 2006	Three-way crossover	7, m, 23 ± 3	Running between 4 and 10 h per week	Oral prednisolone 20 mg, and Oral prednisolone 20 mg and 4 mg salbutamol	Cycling to exhaustion at 80%–85% of VO_2max_
Arlettaz et al., 2007	Crossover	10, m 20 ± 2	Cycling and/or running three to five times per week	Oral prednisolone 5 mg per day for 1 week	Cycling to exhaustion at 70%–75% of VO_2max_
Arlettaz et al., 2008	Crossover	9, m, 23 ± 4	Cycling and/or running 4–8 h per week	Oral prednisolone 20 mg	Steady-state exercise at 60% of VO_2max_ for 1 h
Casuso et al., 2014	Crossover	17, m, 25 ± 3	Healthy non-smoking	Oral dexamethasone 2 mg morning and evening for 5 days	One-leg kicking to exhaustion 20-m shuttle run 30-m sprint
Collomp et al., 2008	Crossover	8, m, 21 ± 1	Cycling and/or running 2–3 times/week	Oral prednisolone 5 mg daily for 7 days	Cycling to exhaustion at 70%–75% VO_2_peak
Jardim et al., 2007	Crossover	12, m, 30 ± 8	Normal post-graduate students	Inhaled flunisolide, 1 mg daily for 4 weeks.	Handgrip strength Handgrip endurance
Kuipers et al., 2008	Parallel	28, m, 24 ± 6	Well-trained endurance athletes	Inhaled budesonide 400 µg morning and evening for 2 and 4 weeks	Incremental cycling test
Le Panse et al., 2009	Crossover	9, f, 20 ± 1	Cycling and/or running two to three times per week	Oral prednisone 50 mg per day for 1 week	Cycling to exhaustion at 70%–75% of VO_2max_
Nordsborg et al., 2008	Crossover	9, m, 24 ± 4	Recreationally active	Oral dexamethasone 2 × 2 mg	Kicking to exhaustion over ∼350 and 110 s
Petrides et al., 1997	Three-way crossover	7/8, m 30 ± 3/28 ± 3	Running 24–40 km per week	Oral dexamethasone 4 mg Oral hydrocortisone 100 mg	10 × 30 s running at as peed corresponding to 90% VO_2max_ alternated with 30 s of rest
Petrides et al., 1994	Crossover	11, m, 28 ± 7	Running 24–40 km per week	Oral dexamethasone 4 mg	10 × 30 s running at as peed corresponding to 90% VO_2max_ alternated with 30 s of rest
Robertson et al., 2016	Crossover	10, m, 40 ± 10	Cyclists and triathletes training minimum 2 h per week	Oral cortisol 50 mg	30 min cycling time trial with sprints
Short et al., 2004	Crossover	3/3, m/f, 30 ± 7	Healthy people	Oral prednisone, 0.5 mg/kg*d for 6 days	Handgrip strength Chest press Biceps curl
Zorgati et al., 2014	Crossover	10, m, 21 ± 1	Physically active 3-4 times per week	Oral prednisone 60 mg per day for 1 week	4 × 30 s hopping at a 100 hop per min

m, male; f, female; *n*, number of participants; SD, standard deviation; VO_2max_, maximal oxygen consumption.

### 3.2. Risk of bias

**Figure 2:**
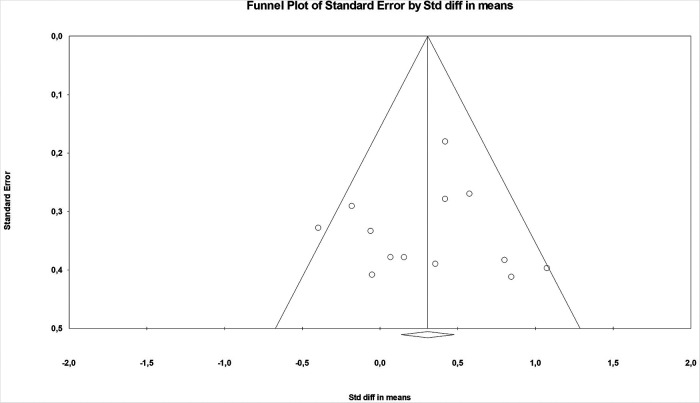
Funnel plot for the effect of glucocorticoids on maximal performance. The plot indicate no proclivity for publishing studies that found a significant effect of glucocorticoids.

Two studies were considered to have a high risk of bias due to low validity of the outcome measure and the nine studies with prolonged administration of glucocorticoids had some concerns related to bias due to no analysis of carry-over effect or data on compliance (Table 2). None of the included studies indicated if the study protocol was pre-registered or not. The funnel plot did not indicate any publication bias (Figure 2) and the Begg and Mazumdar rank correlation test found no publication bias, with a one tailed *p*-value of 0.165 and a classic fail-safe of 26.

**Table 2:** Risk of bias, mean/SD as an assessment of skewness and the number of participants required to obtain alpha 0.05 and beta 0.2.

Study, (author, year)	Risk of bias arising from the randomization process	Risk of bias arising from period and carryover effects	Risk of bias due to deviations from the intended interventions	Risk of bias due to missing outcome data	Risk of bias in measurement of the outcome	Risk of bias in selection of the reported result	Total risk of bias	Baseline mean/SD	Sample for alpha 0.05 and beta 0.2
Arlettaz et al., 2008	Low Risk	Low risk	Low Risk	Low Risk	Low Risk	Low Risk	Low risk	2.9	118
Arlettaz et al., 2006	Low Risk	Low risk	Low Risk	Low Risk	Low Risk	Low Risk	Low risk	3.3	2,743
Arlettaz et al., 2007	Low Risk	Some conserns	Some conserns	Low Risk	Low Risk	Low Risk	Some concerns	2.7	18
Arlettaz et al., 2008	Low Risk	Low risk	Low Risk	Low Risk	Low Risk	Low Risk	Low risk	22.2	118
Casuso et al., 2014	Low Risk	Some conserns	Some conserns	Low Risk	Low Risk	Some conserns	Some concerns	45	16
Collomp et al., 2008	Low Risk	Some conserns	Some conserns	Low Risk	Low Risk	Some conserns	Some concerns	2.2	1,733
Jardim et al., 2007	Low Risk	Some conserns	Some conserns	Low Risk	High Risk	Some conserns	High risk	16.3	141
Kuipers et al., 2008	Low Risk	NA	Some conserns	Low Risk	Low Risk	Low Risk	Some concerns	10.4	1,343
Le Panse et al., 2009	Low Risk	Low risk	Some conserns	Low Risk	Low Risk	Low Risk	Some concerns	2.6	29
Nordsborg et al., 2008	Low Risk	Low risk	Low risk	Some sonserns	Low Risk	Some conserns	Some concerns	2.6	5,086
Petrides et al., 1997	Low Risk	Low risk	Low risk	Low Risk	Low Risk	Low Risk	Low risk	13.8	106
Petrides et al., 1994	Low Risk	Low risk	Low risk	Low Risk	Low Risk	Low Risk	Low risk	10.1	1,570
Robertson et al., 2016	Low Risk	Low risk	Low risk	Low Risk	Low Risk	Some conserns	Some concerns	5.2	82
Short et al., 2004	Low Risk	Some conserns	Some conserns	Low Risk	Low Risk	Some conserns	Some concerns	2.4	6,872
Zorgati et al., 2014	Low Risk	Some conserns	Some conserns	Low Risk	High Risk	Low Risk	High risk	4.2	1,334

### 3.3. Effect of glucocorticoids

**Figure 3:**
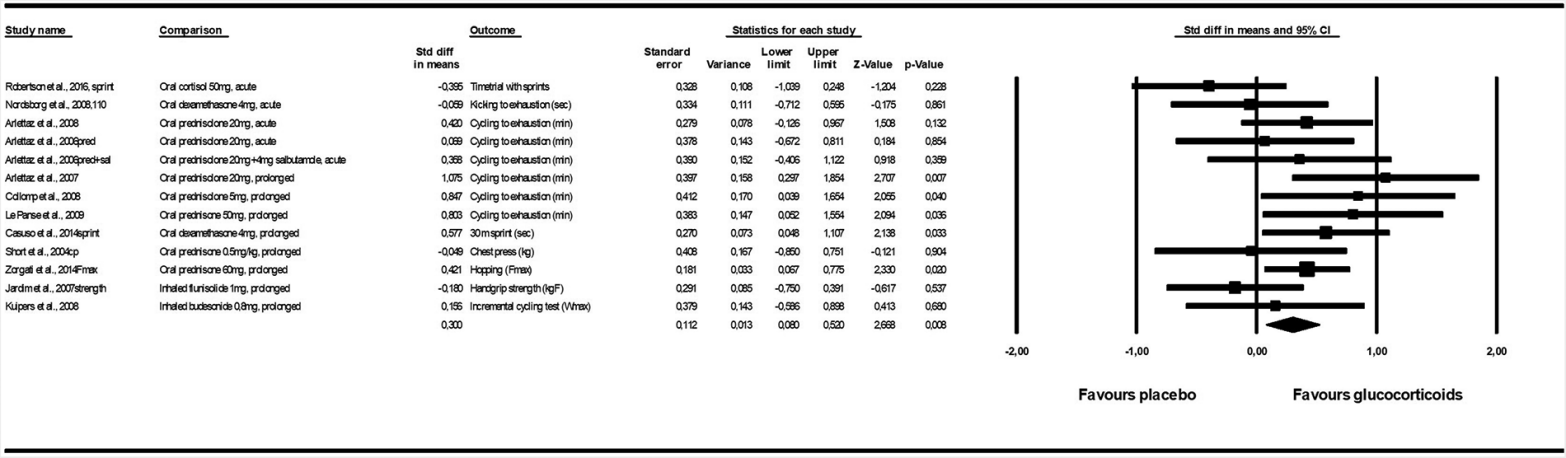
Forest plot fot the effect of glucocorticoids on maximal performance.

Glucocorticoids improved maximal physical performance compared to placebo (SDM 0.300, 95% CI 0.080 to 0.520). The SDM for the 13 included comparisons was not heterogeneous (*I*2^ ^= 35%, *p* = 0.099) (Figure 3). Sensitivity analysis excluding the two studies with high risk of bias showed similar effect (SDM 0.349, 95% CI 0.071 to 0.626). With meta regression we found that duration of treatment, route of administration, and type of exercise did not (*p* > 0.124) influence the SDM. In stratified analysis prolonged treatment and oral ingestion improved physical performance (*p* = 0.003). Acute treatment and inhalation had no effect on physical performance (*p* > 0.564), Sensitivity analysis with high risk of bias studies removed or only one treatment per control group, showed similar effect as the full analysis with SDM 0.334, 95% CI 0.075 to 0.592 and SDM 0.296 0.059 to 0.532, respectively (Table 3). Sensitivity analysis excluding the six comparisons with less than 10 data pairs indicated no effect of glucocorticoids on physical performance (*p* = 0.070). Glucocorticoids improved aerobic performance (SDM 0.348, 95% CI 0.129 to 0.567). Three comparisons tested the effect of glucocorticoids on maximal anaerobic performance and meta-analysis of the comparisons showed no effect (*p* = 0.573) on physical performance. The effect remained not statistically significant after including the two studies measuring anaerobic performance within an aerobic performance test (*p* = 0.491) and when all anaerobic performance outcomes in the included studies (also multiple outcomes from the same study) were meta analysed (*p* = 0.177) (Table 3). We found no effect of glucocorticoids on energy expenditure during submaximal performance (SDM 0.-332, 95% CI −0.785 to 0.121) (Supplementary Figure S1).

**Table 3:** Meta-analysis for the effect of glucocorticoids on various pysicical performance outcomes and maximal performance stratified by duration of treatment and route of administration.

Outcome/strata	Comparisons/subjects (*n*)	Meta-analysis			Test of hetrogeneity	
		SDM	95% CI	*p*-value	*I*2 (%)	*p*-value
Maximal physical performance	13/140^a^^,^^b^	0.300	0.080 to 0.520	0.008	35	0.099
Aerobic performance	12/134^a^^,^^b^	0.371	0.173 to 0.569	<0.001	0	0.585
Anaerobic performance	3/35	0.146	−0.362 to 0.653	0.573	50	0.135
Anaerobic performance + anaerobic performance within aerobic performance	5/55	0.129	−0.238 to 0.396	0.491	55	0.066
Anaerobic performance + anaerobic performance within aerobic performance + multiple outcomes from one study	7/55	0.104	−0.185 to 0.392	0.481	37	0.143
Submaximal performance	4/35	−0.332	−0.785 to 0.121	0.151	5	0.370
Acute treatment	5/40^a^	0.091	−0.202 to 0.392	0.565	6	0.372
Prolonged treatment	8/100^b^	0.428	0.148 to 0.709	0.003	37	0.131
Oral ingestion	11/100^a^	0.361	0.124 to 0.598	0.003	34	0.124
Inhalation	2/40^b^	−0.055	−0.507 to 0.397	0.812	0	0.482

Maximal performance: Outcomes from pysical test with maximal effort. Aerobic performance outcomes from test with maximal effort lasting >1 min. Anaerobic performance: outcomes from test with maximal effort lasting <1 min. Acute treatment: treatment administred at on timepoint, Prolonged treatment: treatment administred over several days.

^a^ Inclucing a threeway crossover trial with 7 subjects.

^b^ Including a paralell trial with 28 subjects.

### 3.4. Skewness and sample size

A baseline performance mean/SD < 2 indicates the outcomes were not skewed in any included study and one study had enough subjects to obtain an alfa of 0.05 and a beta of 0.2 (Table 2).

## 4. Discussion

This systematic review with meta-analysis of RCTs that examined the effect of glucocorticoids on physical performance and comprised 25 different comparisons of performance between glucocorticoids and performance. Two studies had high risk of bias due to low validity of the outcome measure and additionally nine studies had some concern related to bias, as they did not report compliance or performed analysis for carryover effect in trials with prolonged glucocorticoid treatment. Our study extends previous reviews by performing a systematic search, a meta-analysis and including three studies [investigating maximal (29, 33) and submaximal (19) performance] not included in recent reviews on the topic (5, 10). Our analysis demonstrates that oral glucocorticoids improve maximal performance and aerobic performance, especially during prolonged use. Use of glucocorticoids did not influence energy expenditure during submaximal aerobic exercise, and we did not find any effect of glucocorticoids on anaerobic performance.

To our knowledge, no other studies have systematically searched for studies investigating the effect of glucocorticoids on physical performance in healthy subjects, and we are not aware of any meta-analysis performed on the topic. In a narrative review from 2016, Collomp et al. (5) aimed to summarize the current knowledge on the ergogenic effects of glucocorticoids in humans. They reported that an ergogenic effect (on endurance exercise) of short-term systemic glucocorticoids has been clearly demonstrated, and that short-term (4.5 days) and long-term effects (4 weeks) glucocorticoid intake had no effect on VO_2_-max or maximal power output during graded exercise protocols. They also have field test as a performance test category, but refer to only the study by Casuso et al. (27) reporting improved shuttle run performance, but no change in sprint performance. Our findings support and strengthen Collomp et al.’s (5) conclusion with regard to endurance exercise as we also find that prolonged glucocorticoid treatment improves aerobic performance. However, in contrast to Collomp et al. (5) we included both graded exercise tests and field tests (lasting 1 min or more) in our definition of aerobic performance and thus, more studies which added statistical power to our analysis. We found no effect of glucocorticoids on anaerobic performance when we analysed according to our study protocol, however we only included three studies testing anaerobic performance. To increase the statistical power, we also included anaerobic performance within aerobic tests and multiple anaerobic tests from the same study, but still no effect of glucocorticoids was evident. This approach may decrease the validly of the analysis as anaerobic performance within aerobic tests may test other abilities than anaerobic tests and meta-analysing multiple outcomes from the same subjects and intervention is not recommended by the Cochrane Handbook (36). Collomp et al. (5) conclude that it is not clear if glucocorticoids improve performance during short-intense exercise. This conclusion is still valid as only a few studies have investigated the effect of glucocorticoids on anaerobic/short intense exercise. However, when we meta-analyse all available evidence, it seems as glucocorticoids do not improve anaerobic performance. Glucocorticoids did not affected submaximal performance by increasing total energy expended and/or VO_2max_ at a fixed load but this conclusion should also be interfered with cation as the analysis includes only 35 subjects which provides limited the statistical power.

### 4.1. Bias

The funnel plot and the Begg and Mazumdar rank correlation test showed no publication bias and the classic fail-safe of 26. This indicates that the results do not influence whether the studies are published or not and that there would need to be 26 unpublished studies to change the conclusion from the meta-analysis, which is highly unlikely. Two studies were found to have a high risk of bias due to low validity of the performance tests, however omitting these studies from the analysis did not influence the result. Assessing bias is partly subjective also when using a ROB2, the standardised tool recommended by PRISMA (38). We chose to classify all studies with prolonged treatment to have some concerns related to bias, as they provided no information about compliance and no analysis for crossover effect. We could have classified the remaining studies as some concern related to bias as well, as none of the included studies had a preregistered study protocol. But we chose not to, as many of the studies were performed prior to registration was recommended and the outcomes in the present study must have been planned in advance as all outcomes rely on physical tests. Six studies (five testing maximal and one testing submaximal performance) including seven comparisons had included less than 10 data pairs. Low sample size in individual studies in meta-analysis may introduce sparse data bias (39), and thus we performed sensitivity analysis excluding all studies with less than 10 data pairs. The analysis showed no effect of glucocorticoids on physical performance. This was not necessarily related to sparse data bias but may also have been due to low statistical power as we excluded more than half of the comparisons from the main analysis. For this analysis we were left with six comparisons, of which four had anaerobic performance as outcome, and our previous analysis showed that glucocorticoid did not improve anaerobic performance. Only one for the included studies had enough subjects to obtain an alfa of 0.05 and a beta of 0.2, indicating that most studies were underpowered. Normal distribution of the outcome variables in the included studies is an important assumption for meta-analysis of continuous data. No study reported if the outcome data were normally distributed, but all studies had baseline mean/SD > 2, indicating that the outcome variables of the individual studies were not skewed.

### 4.2. Strength and limitations

A strength of the present study is the systematic search for literature in multiple databases and independent selection of studies by two authors. It is therefore likely that we identified all relevant studies. We included only blinded RCTs, which are considered the gold standard for experimental study design. We used SDM as the outcome for the meta-analysis enabling us to include al studies investigating maximal physical performance in one meta-analysis. This resulted in the highest possible sample size and statistical power. Further we performed stratified analysis and meta regression because if subcategories with opposite response to glucocorticoids are included in one meta-analysis the responses may be evened out.

The study is weakened by relatively few studies with few subjects and large variation in population, type of glucocorticoid, dose, duration of treatment and outcome measures as it is likely that the effect of glucocorticoids may depend on population, type, dose, duration of treatment and type of performance. Splitting the studies into subcategories is also a limitation as the statistical power is further reduced. Categories with few studies/subjects usually have larger uncertainties in the effect estimates and it is difficult to interpret if the lack of statistically significant effect is due to no real effect or to low statistical power to reveal the real effect. Another weakness in the present study is that performance is assessed with laboratory tests, not identical to actual athletic competition. Reliable, sensitive and valid test protocols are important and the reliability, sensitivity and validity of several of the included performance tests are questionable. Test protocols should test important athletic qualities and closed end test are recommended over open ended tests due to better reliability, and the coefficient of variations reported to decrease with increased intensity or decreased duration (40). The fitness level of the subjects included in the present study varied from untrained subjects to well-trained endurance athletes, however we were not able to control for the effect of fitness even if physical fitness is known to confound the effect of other WADA prohibited drugs on physical performance (41). Meta-analysis assumes independence between the included studies. In the present study we included the same subject twice in the meta-analysis if they participated in three way crossover trials with two different glucocorticoid interventions. To assess this potential bias, we performed the meta-analysis without one study arm in the three way crossover included in the main analysis, and the result was practically the same as when all relevant comparisons were included. There is also a possibility that the same subjects are included in different studies, but we were not able to control for this. No studies reported the correlation coefficient between the tests with and without glucocorticoids thus the correlation coefficient was set to 0.5 according to previous meta-analysis with performance tests and probably a conservative estimate (42). The present study includes several single blinded RCTs where the investigators probably knew if the subject received treatment or placebo. This lack of blinding may allow the investigator to treat the subjects systematically different during the glucocorticoid and placebo trial. This possible difference in the way the investigators interact with the subjects may lead to a systematic difference in performance. Based on the previously mentioned limitations the findings should be interpreted with caution, especially the results from subgroup analysis due to few subjects and low statistical power. However, there is consistency in the results demonstrating that glucocorticoids improve aerobic performance.

## 5. Conclusion

The present study summarises the best available scientific evidence and indicates that glucocorticoids improve maximal and aerobic performance but do not affect anaerobic performance in healthy subjects. These results should be of interest to WADA, when revising the anti-doping regulations and planning anti-doping sampling and analysis, and to anyone concerned about fair play.

## Data Availability

The original contributions presented in the study are included in the article/**Supplementary Material**, further inquiries can be directed to the corresponding author.

## References

[1] Global Initiative for Asthma. Global strategy for asthma management and prevention (2019). (Accessed September 26, 2019).

[2] Shah A Mak D Davies AM James SL Botchu R. Musculoskeletal corticosteroid administration: current concepts. Can Assoc Radiol J. (2019) 70(1):29–36. 10.1016/j.carj.2018.11.002 30691559

[3] McKenzie DC Fitch KD. The asthmatic athlete: inhaled beta-2 agonists, sport performance, and doping. Clin J Sport Med. (2011) 21(1):46–50. 10.1097/IAE.0b013e318203c0ef 21200170

[4] Hughes D Vlahovich N Welvaert M Tee N Harcourt P White S Glucocorticoid prescribing habits of sports medicine physicians working in high-performance sport: a 30-nation survey. Br J Sports Med. (2020) 54(7):402–7. 10.1136/bjsports-2019-101175 32024647

[5] Collomp K Arlettaz A Buisson C Lecoq AM Mongongu C. Glucocorticoid administration in athletes: performance, metabolism and detection. Steroids. (2016) 115:193–202. 10.1016/j.steroids.2016.09.008 27643452

[6] Fain JN Cheema P Tichansky DS Madan AK. Stimulation of human omental adipose tissue lipolysis by growth hormone plus dexamethasone. Mol Cell Endocrinol. (2008) 295(1):101–5. 10.1016/j.mce.2008.05.014 18640775

[7] Thomasson R Rieth N Jollin L Amiot V Lasne F Collomp K. Short-term glucocorticoid intake and metabolic responses during long-lasting exercise. Horm Metab Res. (2011) 43(3):216–22. 10.1055/s-0030-1269919 21234852

[8] Duclos M Guinot M Le Bouc Y Cortisol GH. Odd and controversial ideas. Appl Physiol Nutr Metab. (2007) 32(5):895–903. 10.1139/h07-064 18059614

[9] Sapolsky RM Romero LM Munck AU. How do glucocorticoids influence stress responses? Integrating permissive, suppressive, stimulatory, and preparative actions. Endocr Rev. (2000) 21(1):55–89. 10.1210/edrv.21.1.0389 10696570

[10] Duclos M. Evidence on ergogenic action of glucocorticoids as a doping agent risk. Phys Sportsmed. (2010) 38(3):121–7. 10.3810/psm.2010.10.1817 20959705

[11] Strehl C Bijlsma JWJ de Wit M Boers M Caeyers N Cutolo M Defining conditions where long-term glucocorticoid treatment has an acceptably low level of harm to facilitate implementation of existing recommendations: viewpoints from an EULAR task force. Ann Rheum Dis. (2016) 75(6):952–7. 10.1136/annrheumdis-2015-208916 26933146

[12] WADA. The prohibited list. Available at: https://www.wada-ama.org/en/prohibited-list#search-anchor (Accessed March 4, 2022).

[13] Ventura R Daley-Yates P Mazzoni I Collomp K Saugy M Buttgereit F A novel approach to improve detection of glucocorticoid doping in sport with new guidance for physicians prescribing for athletes. Br J Sports Med. (2021) 55(11):631–42. 10.1136/bjsports-2020-103512 33879477

[14] Petrides JS Gold PW Mueller GP Singh A Statakis C Chrousos GP Marked differences in functioning of the hypothalamicpituitary-adrenal axis between groups of men. J Appl Physiol. (1997) 82(6):1979–88. 10.1152/jappl.1997.82.6.1979 9173967

[15] Petrides JS Mueller GP Kalogeras KT Chrousos GP Gold PW Deuster PA. Exercise-induced activation of the hypothalamic-pituitary-adrenal axis: marked differences in the sensitivity to glucocorticoid suppression. J Clin Endocrinol Metab. (1994) 79(2):377–83. 10.1210/jcem.79.2.8045951 8045951

[16] Vernec A Slack A Harcourt PR Budgett R Duclos M Kinahan A Glucocorticoids in elite sport: current status, controversies and innovative management strategies-a narrative review. Br J Sports Med. (2020) 54(1):8. 10.1136/bjsports-2018-100196 31326919 PMC6923944

[17] Tacey A Parker L Yeap BB Joseph J Lim EM Garnham A Single-dose prednisolone alters endocrine and haematologic responses and exercise performance in men. Endocr Connect. (2019) 8(2):111–9. 10.1530/ec-18-0473 30673629 PMC6373622

[18] Gastin PB. Energy system interaction and relative contribution during maximal exercise. Sports Med. (2001) 31(10):725–41. 10.2165/00007256-200131100-00003 11547894

[19] Zorgati H Prieur F Vergniaud T Cottin F Do MC Labsy Z Ergogenic and metabolic effects of oral glucocorticoid intake during repeated bouts of high-intensity exercise. Steroids. (2014) 86:10–5. 10.1016/j.steroids.2014.04.008 24793567

[20] Marquet P Lac G Chassain AP Habrioux G Galen FX. Dexamethasone in resting and exercising men. I. Effects on bioenergetics, minerals, and related hormones. J Appl Physiol. (1999) 87(1):175–82. 10.1152/jappl.1999.87.1.175 10409572

[21] Sterne JAC Savović J Page MJ Elbers RG Blencowe NS Burton I Rob 2: a revised tool for assessing risk of bias in randomised trials. Br Med J. (2019) 366:l4898. 10.1136/bmj.l4898 31462531

[22] Sample size calculator. Available at: https://clincalc.com/stats/SampleSize.aspxUpdate (Accessed May 16, 2019).

[23] Altman DG Bland JM. Detecting skewness from summary information. Br Med J. (1996) 313(7066):1200. 10.1136/bmj.313.7066.1200 PMC2352469 8916759

[24] Arlettaz A Collomp K Portier H Lecoq AM Pelle A de Ceaurriz J. Effects of acute prednisolone intake during intense submaximal exercise. Int J Sports Med. (2006) 27(9):673–9. 10.1055/s-2005-872826 16944396

[25] Arlettaz A Collomp K Portier H Lecoq AM Reith N le Pance B Effects of acute prednisolone administration on exercise endurance and metabolism. Br J Sports Med. (2008) 42(4):250–4. 10.1136/bjsm.2007.039040 17609220

[26] Arlettaz A Portier H Lecoq A-M Rieth N De Ceaurriz J Collomp K. Effects of short-term prednisolone intake during submaximal exercise. Med Sci Sports Exerc. (2007) 39(9):1672–8. 10.1249/mss.0b013e3180dc992c 17805102

[27] Casuso RA Melskens L Bruhn T Secher NH Nordsborg NB. Glucocorticoids improve high-intensity exercise performance in humans. Eur J Appl Physiol. (2014) 114(2):419–24. 10.1007/s00421-013-2784-7 24327175

[28] Collomp K Arlettaz A Portier H Lecoq AM le Pance B Reith N Short-term glucocorticoid intake combined with intense training on performance and hormonal responses. Br J Sports Med. (2008) 42(12):983–8. 10.1136/bjsm.2007.043083 18048433

[29] Jardim JR Camelier A Corso SD Rodrigues JE. Strength and endurance of the respiratory and handgrip muscles after the use of flunisolide in normal subjects. Respir Med. (2007) 101(7):1594–9. 10.1016/j.rmed.2006.10.017 17509852

[30] Kuipers H Van't Hullenaar GAC Pluim BM Overbeek SE De Hon O Van Breda EJ. Four weeks’ corticosteroid inhalation does not augment maximal power output in endurance athletes. Br J Sports Med. (2008) 42(11):868–71. 10.1136/bjsm.2007.042572 18344386

[31] Le Panse B Thomasson R Jollin L Lecoq AM Amiot V Reith N Short-term glucocorticoid intake improves exercise endurance in healthy recreationally trained women. Eur J Appl Physiol. (2009) 107(4):437–43. 10.1007/s00421-009-1149-8 19669785

[32] Nordsborg N Ovesen J Thomassen M Zangenberg M Jons C Iaia MF Effect of dexamethasone on skeletal muscle Na+, K+ pump subunit specific expression and K+ homeostasis during exercise in humans. J Physiol Mar. (2008) 586(5):1447–59. 10.1113/jphysiol.2007.143073 PMC2375678 18174214

[33] Robertson CV Immink MA Marino FE. Exogenous cortisol administration; effects on risk taking behavior, exercise performance, and physiological and neurophysiological responses. Front Physiol. (2016) 7:640. 10.3389/fphys.2016.00640 28082908 PMC5186798

[34] Short KR Nygren J Bigelow ML Nair KS. Effect of short-term prednisone use on blood flow, muscle protein metabolism, and function. J Clin Endocrinol Metab. (2004) 89(12):6198–207. 10.1210/jc.2004-0908 15579778

[35] Arlettaz A Portier H Lecoq AM Labsy Z De Ceaurriz J Coiiomp K. Effects of acute prednisolone intake on substrate utilization during submaximal exercise. Int J Sports Med. (2008) 29(1):21–6. 10.1055/s-2007-964994 17614029

[36] Petrides JS Mueller GP Kalogeras KT Chrousos GP Gold PW Deuster PA. Exercise-induced activation of the hypothalamic-pituitary-adrenal axis: marked differences in the sensitivity to glucocorticoid suppression. J Clin Endocrinol Metab. (1994) 79(2):377–83. 10.1210/jcem.79.2.8045951 8045951

[37] Higgins J Thomas J Chandler J Cumpston M Li T Page M Cochrane handbook for systematic reviews of interventions version 6.2. Cochrane (2021). Available at: www.training.cochrane.org/handbook.

[38] Page MJ McKenzie JE Bossuyt PM Boutron I Hoffmann TC Mulrow CD The PRISMA 2020 statement: an updated guideline for reporting systematic reviews. Br Med J. (2021) 372:n71. 10.1136/bmj.n71 33782057 PMC8005924

[39] Greenland S Mansournia MA Altman DG. Sparse data bias: a problem hiding in plain sight. Br Med J. (2016) 352:i1981. 10.1136/bmj.i1981%JBMJ 27121591

[40] Currell K Jeukendrup AE. Validity, reliability and sensitivity of measures of sporting performance. Sports Med. (2008) 38(4):297–316. 10.2165/00007256-200838040-00003 18348590

[41] Price OJ Hull JH Backer V Hostrup M Ansley L. The impact of exercise-induced bronchoconstriction on athletic performance: a systematic review. Sports Med. (2014) 44(12):1749–61. 10.1007/s40279-014-0238-y 25129699

[42] Riiser A Stensrud T Stang J Andersen LB. Can beta2-agonists have an ergogenic effect on strength, sprint or power performance? Systematic review and meta-analysis of RCTs. Br J Sports Med. (2020) 54(22):1351–9. 10.1136/bjsports-2019-100708 32747344

