# Supplementary material for: Glucocorticoids and physical performance: A systematic review with meta-analysis of randomized controlled trials
Source: Front Sports Act Living. 2023 Mar 23;5:1108062. doi: 10.3389/fspor.2023.1108062 (PMC10076788; doi:10.3389/fspor.2023.1108062)
Supplement: Supplementary file 1 [file Table1.docx]

Supplementary table 1. Full search strategy in Web of Science, Skopus, Embase and SportDiscus

| **Database** | Medline |
| --- | --- |
| **Search date** | 25.03.2021 |
| **Search** | Database: Ovid MEDLINE(R) and Epub Ahead of Print, In-Process, In-Data-Review & Other Non-Indexed Citations and Daily <1946 to March 24, 2021>  Search Strategy:  --------------------------------------------------------------------------------  1 exp Healthy Volunteers/ (20709)  2 health*.ti,ab. (2903844)  3 exp Athletes/ (14254)  4 athlete*.ti,ab. (54662)  5 or/1-4 (2956910)  6 exp Glucocorticoids/ (195536)  7 (glucocorticoid* or betamethasone* or beclomethasone* or budesonide* or ciclesonide* or cortisone* or cortivazol* or deflazacort* or dexamethasone* or diflucortolone* or Flunisolide* or Fluticason* or Hydrocortisone* or Methylprednisolone* or mometason* or Prednisolone* or Prednisone* or Triamcinolone Acetonide*).ti,ab. (217107)  8 or/6-7 (307682)  9 exp Athletic Performance/ (56312)  10 (exhaustion or power or endurance or strength or aerobe* or anaerobe* or exercise or athletic performance).ti,ab. (903761)  11 or/9-10 (926590)  12 (random* and (controlled or control or placebo or versus or vs or group or groups or comparison or compared or arm or arms or crossover or cross-over) and (trial or study)).ti,ab. (712764)  13 ((single or double or triple) and (masked or blind*)).ti,ab. (195281)  14 exp Randomized Controlled Trials as Topic/ (145110)  15 exp Randomized Controlled Trial/ (526801)  16 or/12-15 (1029100)  17 exp Patients/ (68526)  18 patient*.ti,ab. (7015977)  19 or/17-18 (7040182)  20 5 and 8 and 11 and 16 (172)  21 20 not 19 (83)  *************************** |
| **Records identified** | 83 (before elimination of duplicates) |

| **Database** | SportDiscus |
| --- | --- |
| **Search date** | 25.03.2021 |
| **Search** | \| **#** \| **Query** \| **Limiters/Expanders** \| **Last Run Via** \| **Results** \| \| --- \| --- \| --- \| --- \| --- \| \| S1 \| TI ( health* OR athlete* ) OR AB ( health* OR athlete* ) OR KW ( health* OR athlete* ) \| Expanders - Apply equivalent subjects  Search modes - Boolean/Phrase \| Interface - EBSCOhost Research Databases  Search Screen - Advanced Search  Database - SPORTDiscus with Full Text \| 325,818 \| \| S2 \| DE "ATHLETES" \| Expanders - Apply equivalent subjects  Search modes - Boolean/Phrase \| Interface - EBSCOhost Research Databases  Search Screen - Advanced Search  Database - SPORTDiscus with Full Text \| 51,867 \| \| S3 \| S1 OR S2 \| Expanders - Apply equivalent subjects  Search modes - Boolean/Phrase \| Interface - EBSCOhost Research Databases  Search Screen - Advanced Search  Database - SPORTDiscus with Full Text \| 348,644 \| \| S4 \| DE "GLUCOCORTICOIDS" \| Expanders - Apply equivalent subjects  Search modes - Boolean/Phrase \| Interface - EBSCOhost Research Databases  Search Screen - Advanced Search  Database - SPORTDiscus with Full Text \| 554 \| \| S5 \| TI ( glucocorticoid* or betamethasone* or beclomethasone* or budesonide* or ciclesonide* or cortisone* or cortivazol* or deflazacort* or dexamethasone* or diflucortolone* or Flunisolide* or Fluticason* or Hydrocortisone* or Methylprednisolone* or mometason* or Prednisolone* or Prednisone* or "Triamcinolone Acetonide*" ) OR AB ( glucocorticoid* or betamethasone* or beclomethasone* or budesonide* or ciclesonide* or cortisone* or cortivazol* or deflazacort* or dexamethasone* or diflucortolone* or Flunisolide* or Fluticason* or Hydrocortisone* or Methylprednisolone* or mometason* or Prednisolone* or Prednisone* or "Triamcinolone Acetonide*" ) OR KW ( glucocorticoid* or betamethasone* or beclomethasone* or budesonide* or ciclesonide* or cortisone* or cortivazol* or deflazacort* or dexamethasone* or diflucortolone* or Flunisolide* or Fluticason* or Hydrocortisone* or Methylprednisolone* or mometason* or Prednisolone* or Prednisone* or "Triamcinolone Acetonide*" ) \| Expanders - Apply equivalent subjects  Search modes - Boolean/Phrase \| Interface - EBSCOhost Research Databases  Search Screen - Advanced Search  Database - SPORTDiscus with Full Text \| 2,355 \| \| S6 \| S4 OR S5 \| Expanders - Apply equivalent subjects  Search modes - Boolean/Phrase \| Interface - EBSCOhost Research Databases  Search Screen - Advanced Search  Database - SPORTDiscus with Full Text \| 2,465 \| \| S7 \| TI ( exhaustion or power or endurance or strength or aerobe* or anaerobe* or exercise or "athletic performance" ) OR AB ( exhaustion or power or endurance or strength or aerobe* or anaerobe* or exercise or "athletic performance" ) OR KW ( exhaustion or power or endurance or strength or aerobe* or anaerobe* or exercise or "athletic performance" ) \| Expanders - Apply equivalent subjects  Search modes - Boolean/Phrase \| Interface - EBSCOhost Research Databases  Search Screen - Advanced Search  Database - SPORTDiscus with Full Text \| 251,737 \| \| S8 \| TI ( random* and (controlled or control or placebo or versus or vs or group or groups or comparison or compared or arm or arms or crossover or cross-over) and (trial or study) ) OR AB ( random* and (controlled or control or placebo or versus or vs or group or groups or comparison or compared or arm or arms or crossover or cross-over) and (trial or study) ) OR KW ( random* and (controlled or control or placebo or versus or vs or group or groups or comparison or compared or arm or arms or crossover or cross-over) and (trial or study) ) \| Expanders - Apply equivalent subjects  Search modes - Boolean/Phrase \| Interface - EBSCOhost Research Databases  Search Screen - Advanced Search  Database - SPORTDiscus with Full Text \| 42,545 \| \| S9 \| TI ( (single or double or triple) and (masked or blind*) ) OR AB ( (single or double or triple) and (masked or blind*) ) OR KW ( (single or double or triple) and (masked or blind*) ) \| Expanders - Apply equivalent subjects  Search modes - Boolean/Phrase \| Interface - EBSCOhost Research Databases  Search Screen - Advanced Search  Database - SPORTDiscus with Full Text \| 9,494 \| \| S10 \| S8 OR S9 \| Expanders - Apply equivalent subjects  Search modes - Boolean/Phrase \| Interface - EBSCOhost Research Databases  Search Screen - Advanced Search  Database - SPORTDiscus with Full Text \| 45,349 \| \| S11 \| TI patient* OR AB patient* OR KW patient* \| Expanders - Apply equivalent subjects  Search modes - Boolean/Phrase \| Interface - EBSCOhost Research Databases  Search Screen - Advanced Search  Database - SPORTDiscus with Full Text \| 135,455 \| \| S12 \| S3 AND S6 AND S7 AND S10 \| Expanders - Apply equivalent subjects  Search modes - Boolean/Phrase \| Interface - EBSCOhost Research Databases  Search Screen - Advanced Search  Database - SPORTDiscus with Full Text \| 20 \| |
| **Records** | 20 (before elimination of duplicates) |
| **Comment** | It is not possible to use NOT in this database (thus it is not included in the search) |

| **Database** | Embase |
| --- | --- |
| **Search date** | 25.03.2021 |
| **Search** | Database: Embase <1974 to 2021 Week 11>  Search Strategy:  --------------------------------------------------------------------------------  1 exp normal human/ (748630)  2 health*.ti,ab. (3871225)  3 exp athlete/ (61980)  4 athlete*.ti,ab. (65885)  5 or/1-4 (4432946)  6 exp glucocorticoid/ (739939)  7 (glucocorticoid* or betamethasone* or beclomethasone* or budesonide* or ciclesonide* or cortisone* or cortivazol* or deflazacort* or dexamethasone* or diflucortolone* or Flunisolide* or Fluticason* or Hydrocortisone* or Methylprednisolone* or mometason* or Prednisolone* or Prednisone* or Triamcinolone Acetonide*).ti,ab. (300514)  8 or/6-7 (782394)  9 exp athletic performance/ (12399)  10 (exhaustion or power or endurance or strength or aerobe* or anaerobe* or exercise or athletic performance).ti,ab. (1105182)  11 or/9-10 (1110860)  12 (random* and (controlled or control or placebo or versus or vs or group or groups or comparison or compared or arm or arms or crossover or cross-over) and (trial or study)).ti,ab. (1027968)  13 ((single or double or triple) and (masked or blind*)).ti,ab. (276930)  14 exp randomized controlled trial/ (654631)  15 or/12-14 (1291522)  16 exp patient/ (2577658)  17 patient*.ti,ab. (10235588)  18 or/16-17 (10449719)  19 5 and 8 and 11 and 15 (837)  20 19 not 18 (567)  *************************** |
| **Records** | 567 (before elimination of duplicates) |

| **Database** | Web of Science |
| --- | --- |
| **Search date** | 25.03.2021 |
| **Search** | 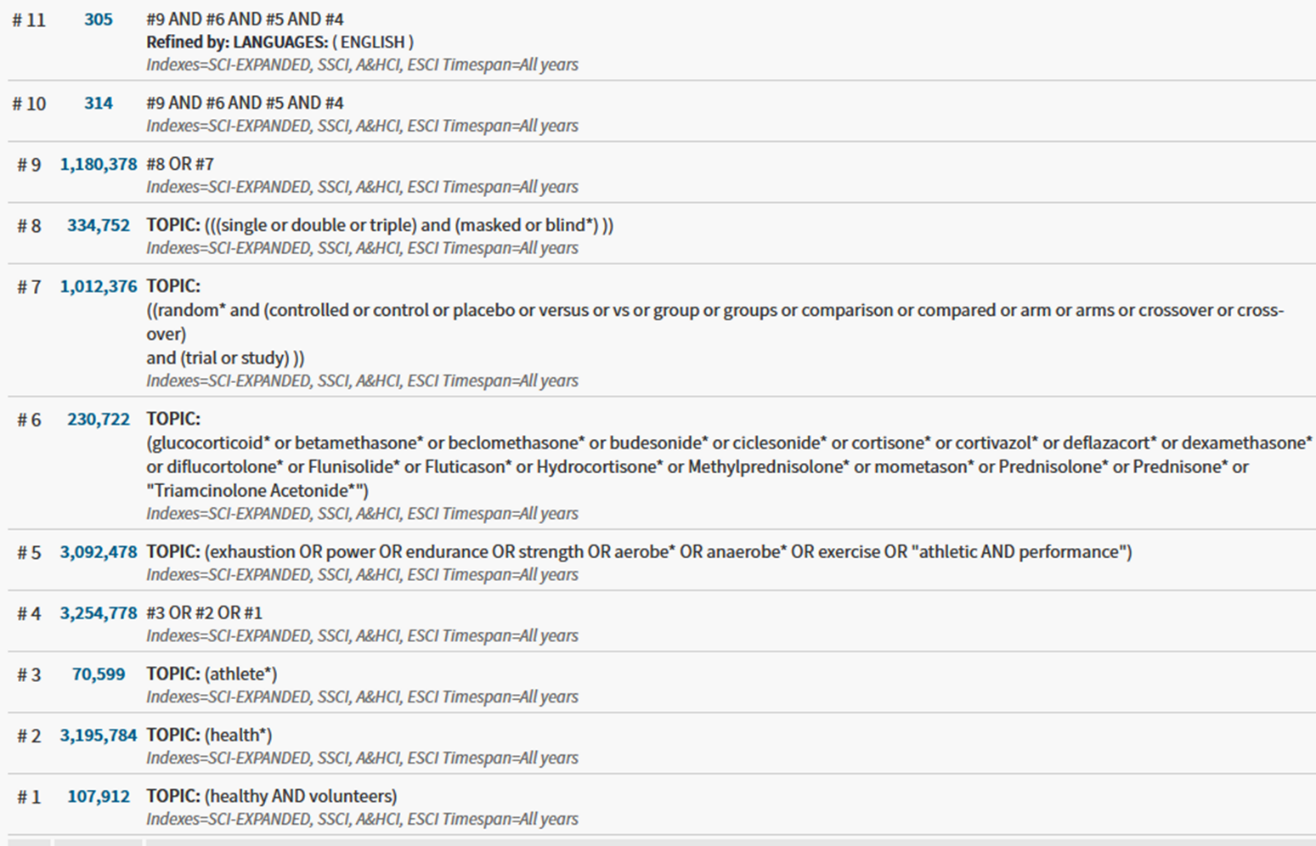 |
| **Records** | 305 (before elimination of duplicates) |

| **Database** | Scopus |
| --- | --- |
| **Search date** | 25.03.2021 |
| **Search** | 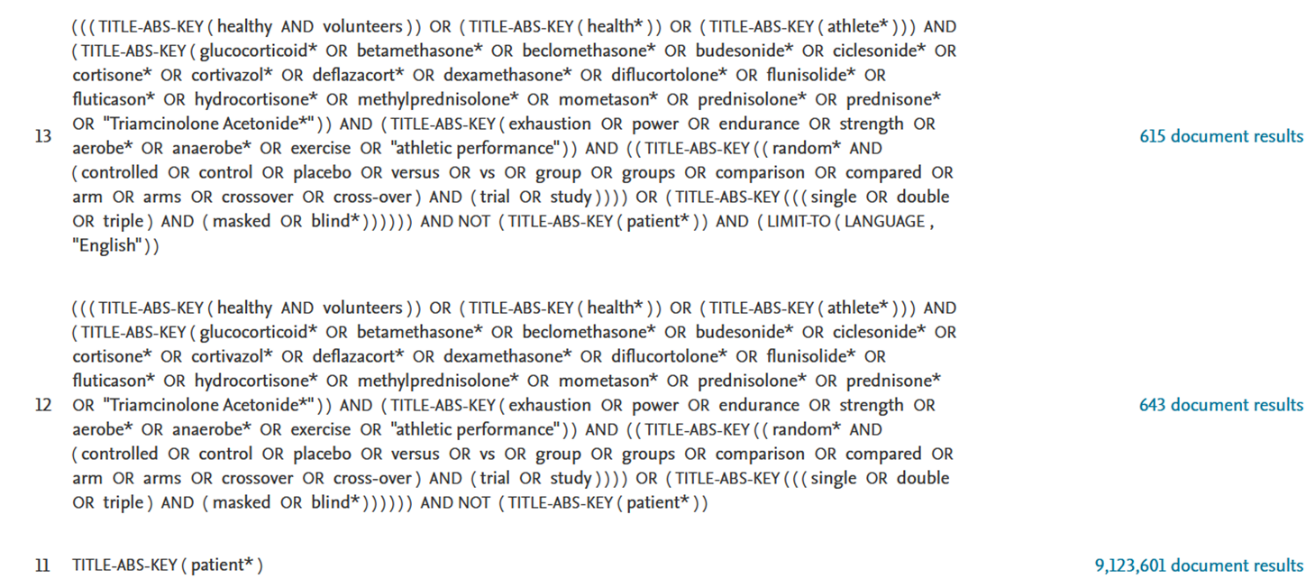  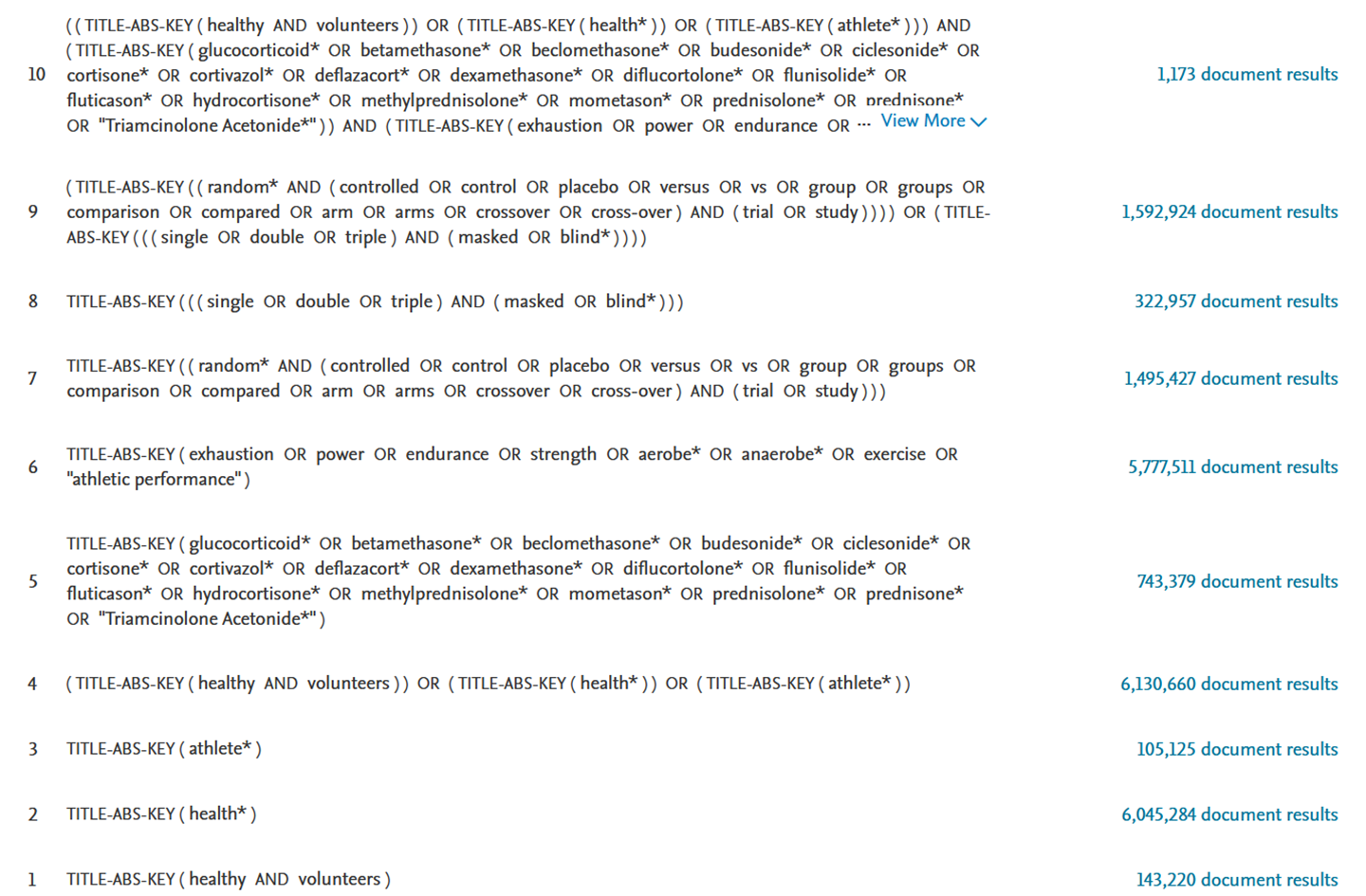 |
| **Records** | 615 (before elimination of duplicates) |

1245 totalt

1077 etter fjerning av duplikater
