# Supplementary figures and images for: Glucocorticoids and physical performance: A systematic review with meta-analysis of randomized controlled trials
Source: Front Sports Act Living. 2023 Mar 23;5:1108062. doi: 10.3389/fspor.2023.1108062 (PMC10076788; doi:10.3389/fspor.2023.1108062)

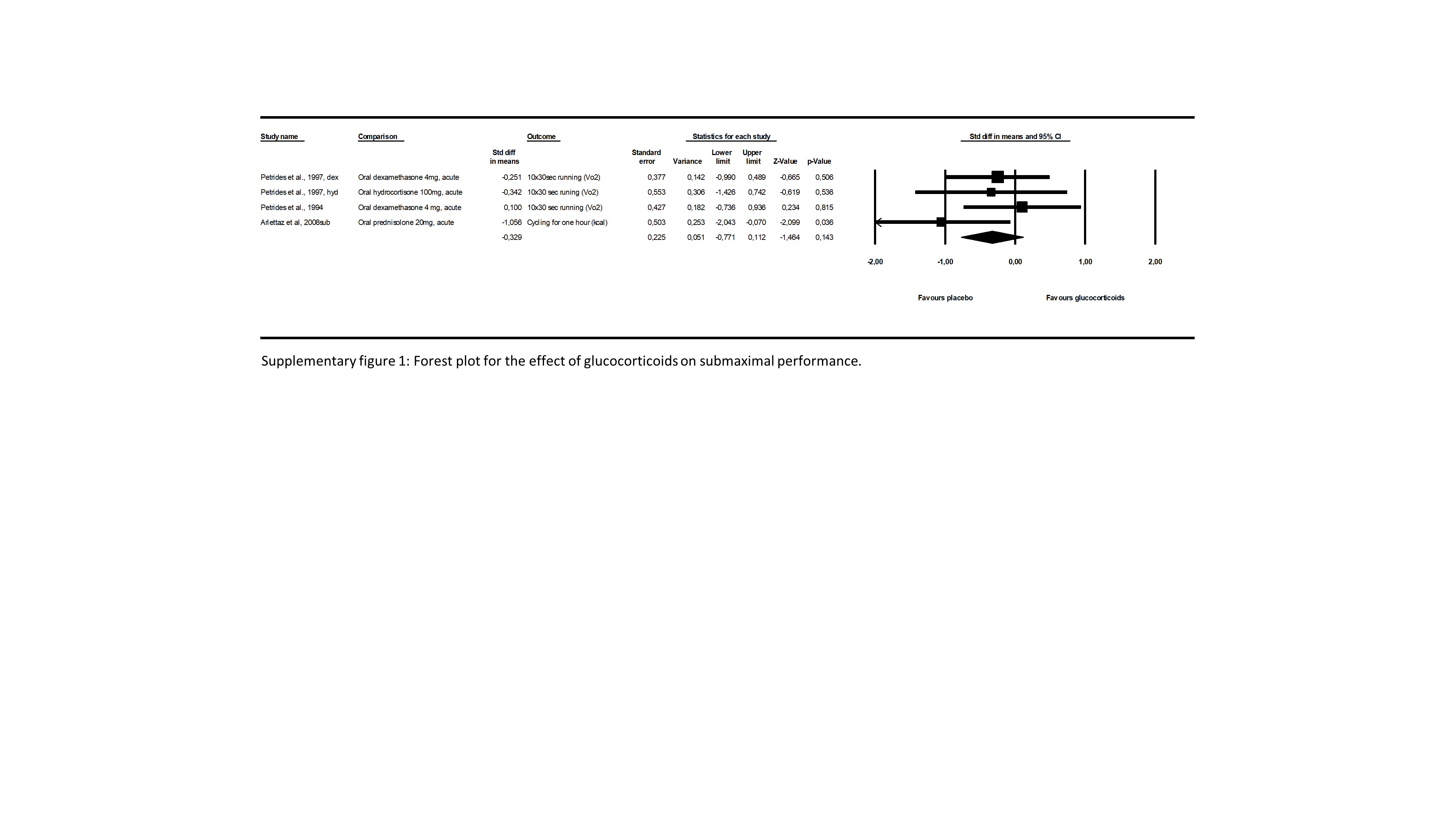

Supplement: Supplementary file 2 [file Image1.jpeg]
